# Insights from the molecular docking analysis of compounds from *Vitex negundoi* with targets from *Klebsiella pneumoniai* causing urinary tract infection

**DOI:** 10.6026/973206300181062

**Published:** 2022-11-30

**Authors:** Preetha SP, Arul Salomee Kamalabai R, Jayachandran KS

**Affiliations:** 1 Department of Bioinformatics, Bharathidasan University, Tiruchirappalli - 620024; 2 Department of Veterinary Pharmacology and Toxicology, Madras Veterinary College, Tamil Nadu Veterinary and Animal Sciences University, Chennai - 600007

**Keywords:** molecular docking, *Vitex negundoi*, *Klebsiella pneumoniai*

## Abstract

Antimicrobial resistance among bacterial strains has emerged out to be a serious threat and contributes to the loss of effectiveness of the common antibiotics. New Delhi metallo-β-lactamase-1 (blaNDM-1) is an enzyme present in several pathogenic bacteria with a high incidence in *Klebsiella pneumoniaie* and plays a crucial role in the development of antibacterial resistance. Mur enzymes are also important alternative drug targets in addition to blaNDM-1 which are crucial for the survival of the bacteria. *Vitex negundoi* is an aromatic medicinaltree with proven antibacterial properties. Fifteen compounds from *V. negundo* were evaluated for their inhibitory effects on the target proteins blaNDM-1, Mur C, Mur E and Mur F of *K. pneumoniae* through molecular docking using the Glide (xp) module of Schrodinger. ADME toxicity was also predicted for all the fifteen compounds in the QikProp module. The docking results revealed that the compounds agnuside, negundoside and isoorientin showed promising inhibitory effects on all four targets blaNDM-1, Mur C, Mur E and Mur F of *K. pneumoniae* with docking scores greater than -7 kcal/mol and reasonable hydrogen bond interactions. The findings of this study provide a lead for developing novel drugs against potent multidrug-resistant *K. pneumoniae*.

## Background:

Urinary Tract Infection (UTI) due to bacteria can occur anywhere in the bladder (Cystitis), kidney (Pyelonephritis), and ureter or in the urethra (Urethritis) [1]. There are several factors responsible for the occurrence of UTI in both men and women of all ages and population, though women are more prone. Nearly 150 million people suffer due to UTI every year [2]. The predominant pathogens responsible for UTI are *E. coli* and *Klebsiella pneumoniaie* which accounts for 60% and 16% of the infection respectively [3]. The other bacteria associated with UTI include *Proteusi*, *Citrobacteri*, *Pseudomonasi*, *Acinetobacter*, *Providencia* and *Enterobacter* [4]. β-lactam antibiotics has been used to treat the bacterial infections by interfering with the peptidoglycan layer in the bacterial cell wall [5]. The pathogens have developed resistance against the β-lactam antibiotics by producing various β-lactamases [6, 7]. New Delhi metallo-β-lactamase-1 (blaNDM-1) is a β-lactamase present in several pathogenic bacteria including *E. coli*, *K. pneumoniae*, *K. oxytoca*, *Enterobacter choacae*, *Citerobacter freundii*, *Morgenella morganii*, *Proteusi*, *Providencia*, *Pseudomonasi aeroginosa* and *Acinetobcter baumanii* which have developed resistance to antibiotics by Horizontal Gene Transfer (HGT)[8,9]. Of this, the infections caused by *K. pneumoniae* have become difficult to treat due to its rapid development of resistance to the available antibiotics [10]. The development of resistance by the pathogens is due to inappropriate use of antibiotics, self-medication with some types of antibiotics, limited laboratory diagnosis and insufficient dosage of the antibiotics [11]. The treatment options for the bacterial strains that carry the blaNDM-1 gene is a great challenge due to their resistance development to most of the available antibiotics. The antimicrobial resistance development has alarmingly increased worldwide [12], which generates an urgent need to explore newer drug molecules and drug targets that could be used to treat infections when the antibiotics become inefficient. Mur enzymes are also important drug targets in addition to blaNDM-1 which are crucial for the survival of the bacteria [13]. So Mur C, Mur E and Mur F of *K. pneumoniae* were selected as alternative targets in addition to blaNDM-1 for this study to overcome antibiotic resistance. *V. negundo* is a medicinal plant with proven antibacterial properties [14- 15] used in traditional medicine and is commonly distributed in tropical and subtropical areas. With this background information, the present study was designed to develop new drug molecules from the leaves of *V. negundo* against the drug targets blaNDM-1, Mur C, Mur E and Mur F present in the pathogenic bacteria *K. pneumoniae*.

## Methods:

## Target retrieval:

Primary sequence and functional information of four targets blaNDM-1, Mur F, Mur E and Mur C of *K. pneumoniae* were retrieved from UniprotKB (http://ca.expasy.org/sprot/) with Accession number: C7C422, A0A081M2L4, A0A169LLY6 and A0A0W8AVX1. blaNDM-1 has its three-dimensional (3D) structure solved and deposited in PDB with ID: 4EYB. For Mur F, Mur E and Mur C protein structures were predicted using online tools.

## Structure prediction:

Comparative modelling of Mur F, Mur E and Mur C (*K. pneumoniae*) were analysed in the following order. Template search was done and validated by homologous search using BLAST tool (http://blast.ncbi.nlm.nih.gov) against PDB database (http://www.rcsb.org/pdb/). Suitable templates were selected based on the highest sequence identity and query coverage. By using the templates, homology models were generated for Mur F, Mur E and Mur C using Swiss model (http://swissmodel.expasy.org/) an automated modelling server and the three-dimensional structures generated were validated through RAMPAGE.

## Protein preparation:

The three-dimensional structures of blaNDM-1, Mur F, Mur E and Mur C were imported individually into maestro and prepared using Protein Preparation Wizard of the Schrodinger suite (Schrodinger, LLC, New York, NY, 2015). The workflow was carried out with default parameters by removing all water molecules and adding polar hydrogen atoms to the parent carbon atoms. All atoms were charged with OPLS 2001 force fields.

## Active site prediction:

The binding sites for blaNDM-1, Mur F, Mur E and Mur C were predicted using SiteMap protocol (Schrodinger, LLC, New York, NY, 2015) which gives information's about the location of the protein active sites, binding site and functional residues involved in protein-ligand interactions through novel search and analysis facilities. The results obtained through this protocol will be helpful for the prediction of receptor-ligand interactions.

## Ligand retrieval and preparation:

Fifteen bioactive compounds namely Negundin A, Negundin B, Indomethacin, Isoorientin, Isovitexin, Lyoniresinol, Mussaenosidic Acid, Agnuside, Negundoside, Pinoresinol, Vitexin, Vitedoamine A,Vitedoin A, Vitedoin B, Vitexicarpin reported from the leaf extract of *V. negundo* plant were found to have anti-bacterial activities [16-18] and their structures were retrieved from PubChem compound database. The obtained structures were imported into the glide window and converted into maestro format. It was then prepared using the LigPrep module (Schrodinger suite, LLC, New York, NY, 2015). The ligands were geometry optimized using OPLS-2005 force field.

## ADME toxicity prediction:

Unfavourable absorption, distribution, metabolism, excretion and toxicity (ADME Toxicity) properties for all the fifteen compounds of *V. negundo* were predicted using QikProp program (Schrodinger suite LLC, New York, NY, 2015), which also analyses the pharmaceutically relevant properties for indispensable lead generation and lead optimization.

## Molecular docking simulation:

To examine the binding conformation of the fifteen *V. negundo* compounds in the active sites of four targets (blaNDM-1, Mur F, Mur E and Mur C), molecular docking analysis was carried out in Glide module (Schrodinger, LLC, New York, NY, 2015). Initially, the docking area was defined by a grid box using receptor-grid generation protocol (Schrodinger, LLC, New York, NY, 2015). The molecular docking experiments were carried out with default parameters (Glide protocol) using XP docking module. The results of the molecular docking gives information about the drug like molecules of *V. negundo* that interact with the putative binding sites blaNDM-1, Mur F, Mur E and Mur C of *K. pneumoniae* . The docking results were interpreted based on docking score, glide energy and number of Hydrogen bond interactions.

## Results:

### Structure prediction:

A high percentage of sequence similarity between the target sequence and template structure produces reliable three-dimensional structure of the proteins. Protein Blast showed that the Crystal structure of *Escherichia coli* Udpmurnac-Tripeptide D-Alanyl-D-Alanine-adding Enzyme (Mur F) at 2.3 Angstrom Resolution (PDB ID: 1GG4) had 82.04% sequence identity for Mur F with 99% query coverage and 0% gaps. The structure of UDP-N-acetylmuramyl tripeptide synthetase from *E. coli* (Mur E) (PDB ID: 1E8C) had 89.27% sequence identity, 99% query coverage and 0% gaps for Mur E and *Escherichia coli* Mur C (PDB ID: 2F00) had 89.98% sequence identity, 99% query coverage and 0% gaps for Mur C. They were identified as suitable templates. In modelling algorithm, if a protein sequence shares more than 30% identity they were structurally similar. This evidence provides 1GG4, 1E8C and 2F00 as appropriate templates for structure prediction of Mur F, Mur E and Mur C through modelling techniques. The three-dimensional structures predicted using Swiss model for Mur F, Mur E and Mur C are shown in Figure 1(see PDF).

### Model validation:

Validation of the models by Phi and Psi angles through Ramachandran plot reveals that the models generated were in a more acceptable range. The overall main chain and side chain parameters of Mur F, Mur E and Mur C were validated through RAMPAGE. It indicates 97.5% residues in the core region, 2.2% of residues in the allowed region and 0.2% residues in the disallowed region for Mur F, 96.5% residues in the core region, 3.1% of residues in the allowed region and 0.4% residues in the disallowed region for Mur E and 96.6% residues in the core region, 2.8% of residues in the allowed region, and 0.6% residues in the disallowed region for Mur C (Figure 2 - see PDF). These results indicate that the predicted structures are of reasonably good structural parameters.

### Active site prediction:

The best site for the docking approach has been chosen based on the site score and hydrophobic / hydrophilic regions predicted using site map protocol. Figure 3(see PDF) shows the binding cavity of the protein structures for blaNDM-1, Mur F, Mur E and Mur C.

### ADME toxicity prediction:

The 15 *V. negundo* compounds were analysed to determine the pharmacokinetic properties using QiKprop module in the Schrodinger suite. The pharmacokinetic properties such as QPPMDCK (Predict apparent MDCK permeability), QplogHERG (predict IC50 value for blockage of HERG K+ channels), QPPcaco (Predict apparent Caco-2 cell permeability), Rules of 5 (number of violations of Lipinski's rule of five), Molecular weight and stars (Descriptor values that fall outside the 95% range of similar value for known drugs) are presented in Table 1(see PDF).

### Molecular docking simulation:

Molecular docking analysis of the *V. negundo* compounds in the active site of blaNDM-1, Mur F, Mur E and Mur C were performed. The receptor grid generation was recorded which indicates good interaction between proteins and the small molecules. The docking results of all the active compounds are reported in Table 2(see PDF). The ranking of the docking results is based on the docking score, glide energy and number of hydrogen bond interactions. The best docked ligand molecules in the active site of blaNDM-1, Mur F, Mur E and Mur C are shown in the Figure 4(see PDF). All the small molecules having interaction with the binding cavity in various conformations differ in their docking score and glide energy. Among all the bioactive compounds, Isoorientin showed the best binding affinity for blaNDM-1 with docking score -7 kcal/mol, glide energy -64.8 and 5 hydrogen bonds. Agnuside showed best binding affinity for Mur F and Mur C with docking score -6.7 kcal/mol and -7.5 kcal/mol, glide energy -59 and -62.5 respectively with 11 hydrogen bonds. Negundoside showed best binding affinity for Mur E with docking score -10.7 kcal/mol, glide energy -81 and 9 hydrogen bonds. Thus agnuside, isoorientin and negundoside showed promising inhibitory effect in the active site of all the target proteins. The results indicate that *V. negundo* derivatives have potential applications towards blaNDM-1, Mur F, Mur E and Mur C of *K. pneumoniae*.

## Discussion:

Antibiotics work by either killing or inhibiting the growth of the bacteria [19]. The indiscriminate use of antibiotics and the rapid development of antibacterial resistance has created a great challenge in the treatment of infections caused by resistant bacteria [12,13, 14, 15, 16, 16, 17, 18, 19, 20]. blaNDM-1 is the gene coding for carbapenemase β-lactamase enzyme released by certain bacteria that inactivates antibiotics with β-lactam ring [21, 22,23]. According to a recent report blaNDM-1 gene was observed in 44 strains of *K. pneumoniae*, 2 strains of *E. coli* and 8 strains of A. baumannii [9]. There are several studies that focus on blaNDM-1 as a target for drug discovery because of its crucial role in resistance development. But targeting only blaNDM-1 and producing modified antibiotics has not stopped the increase in antibacterial resistance. Hence there is an urge to shift the focus to alternative targets that could overcome antibiotic resistance. So Mur C, Mur E and Mur F of *K. pneumoniae* were selected as alternative targets in addition to blaNDM-1 for this study. Mur enzymes are primarily involved in the peptidoglycan biosynthesis essential for the survival of the bacteria. To develop new molecules against the target proteins blaNDM-1, Mur C, Mur E and Mur F of *K. pneumoniae*, an aromatic medicinal tree V.negundo that has been used traditionally owing to its antibacterial properties was selected for the study. Herbal medicines are becoming popular worldwide because of their minimal side effects [24, 25]. Molecular docking simulation was performed in this study to identify the inhibitory effect of *V. negundo* compounds on *K. pneumoniae* proteins that would collapse the survival of the bacteria. Docking was performed using the Glide (xp) module of Schrodinger with fifteen reported compounds of *V. negundo* against the four targets of *K. pneumoniae*. The results revealed that agnuside, isoorientin, and negundoside showed promising inhibitory effect against all the four target proteins of *K. pneumoniae*. analysed. Findings of the present study could provide a lead for developing novel drugs against multidrug resistant *K. pneumoniae*.
